# Association of hand grip strength and bite force on the presence of plaque among adults aged 35–44 years in Mangalore – a cross-sectional study

**DOI:** 10.1186/s13104-025-07406-w

**Published:** 2025-08-06

**Authors:** Shushma Rao B, Ramya Shenoy, Praveen Jodalli, Ashish John Prabhakar, Roma M, Ashwini Rao, Mithun Pai, Avinash B.R

**Affiliations:** 1 Department of Public Health Dentistry, Manipal College of Dental Sciences Mangalore, Manipal Academy of Higher Education, Manipal, 576104 Karnataka India; 2 Department of Physiotherapy, Kasturba Medical College Mangalore, Manipal Academy of Higher Education, Manipal, 576104 Karnataka India; 3 Department of Conservative Dentistry and Endodontics, Manipal College of Dental Sciences Mangalore, Manipal Academy of Higher Education, Manipal, 576104 Karnataka India

**Keywords:** Adult, Bite force, Dental plaque, Handgrip strength, Hand strength, Health and wellbeing, Plaque levels, Sustainable development goals

## Abstract

**Objective:**

To determine the associations of hand grip strength and bite force on presence of plaque in the adult population in Mangalore. The research hypothesis was that hand grip strength, bite force, and plaque scores are associated.

**Results:**

A total of 48 patients were included, among whom 20 (41.7%) were males and 28 (58.3%) were females, with a mean age of 39.8 ± 3.2 years. A significant relationship was observed between plaque score and education level (*p* = 0.043), method of brushing (*p* = 0.006) and frequency of changing toothbrushes (*p* = 0.000). Handgrip strength and bite force on both the left and right sides were significantly associated with sex (p value: hand grip strength on the right side (HG_rt) = 0.000, handgrip strength on the left side (HG_lt) = 0.000, bite force on the right side (BF_rt) = 0.008, bite force on the left side (BF_lt) = 0.007)) and employment status (p value: HG_rt, HG_lt, BF_rt, BF_lt = 0.000). Attrition was associated with handgrip strength alone (p value: HG_rt = 0.002, HG_lt = 0.011). No significant differences were observed between the plaque score and hand grip strength or between the plaque score and bite force on either the left or right side. Regression analysis with the plaque score as the dependent variable revealed no significant results.

**Supplementary Information:**

The online version contains supplementary material available at 10.1186/s13104-025-07406-w.

## Introduction

Dental plaque is defined clinically as a structured, resilient, yellow‒greyish substance that plays a major role in the initiation and development of dental plaque [1, 2]. The future oral health of individuals is closely related to dental plaque development [3]. Maintaining adequate oral hygiene can reduce plaque, leading to the prevention of oral diseases [4].

Plaque removal improves with improved dexterity, according to a study comparing plaque levels and motor abilities [5]. A study conducted by Jae-Hyun Lee revealed that poor oral hygiene behaviour was associated with low hand grip strength [6]. Handgrip strength is a reliable way to measure voluntary muscle strength and is often used as a substitute for overall muscular fitness [7, 8]. Additionally, reduced masticatory performance is associated with decreased grip strength [9].

Masticatory force has been used to evaluate oral function [8, 10], masticatory performance [9] and dental and jaw rehabilitation outcomes [11]. A Japanese study also revealed a positive relationship between bite force and physical fitness [12]. Although biomechanically discrete, the jaw and hand systems share similar brain representations, probably due to the combined need for sensory and motor control for both fine and gross movements in daily activities [11]. A study conducted by Hung-Huey Tsai revealed that the maximum bite force and Plaque Index were inversely correlated [13]. While studies from Korea [6], Japan [12], and Taiwan [13] have addressed hand grip strength, bite force and plaque individually, similar research within the Indian context, including Mangalore, is lacking. No prior study has comprehensively researched the relationships among handgrip strength, bite force and plaque level in a single study.

The aim of this study was to determine the association of hand grip strength and bite force on presence of plaque among adult population in Mangalore. The primary objective was to assess hand grip strength, bite force, and plaque score. The secondary objective was to assess the associations among hand grip strength, bite force and plaque score. The research hypothesis was that hand grip strength, bite force, and plaque scores are associated.

## Materials and methods

### Study design and setting

This cross-sectional type of analytical study was conducted among adults aged 35–44 years in the dental clinic and outreach centers of MCODS Mangalore. This age group is standard for monitoring oral health conditions, as it reflects the full effect of dental caries, severe periodontal involvement and the general effects of care provided. Moreover, the literature has commonly focused on this age group when evaluating handgrip strength, bite force, and dental plaque level [14–16].

### Eligibility criteria

Consenting adults aged 35–44 years who visited the dental clinic and the outreach centers for dental treatment, had not undergone scaling within the past week, and had at least 20 functional teeth with 1st or 2nd molars on either side were included.

Those who were not able to comprehend the instructions or had preexisting medical conditions (diabetes, hypertension, cardiovascular disorders, musculoskeletal disorders, physical disabilities, and osteoarthritis, which were assessed through thorough history recording) that may affect hand grip strength, individuals with temporomandibular joint (TMJ) disorders (pain/tenderness, clicking, deviation and difficulty in mouth opening), severe malocclusion and those who consumed tobacco/alcohol or both were excluded.

TMJ disorders were diagnosed via medical history taking and clinical examination. The participants were asked about their past history or current experience of pain, clicking sounds, or restricted mouth opening. Clinically, tenderness and clicking were assessed by palpating the joint. Difficulty in mouth opening and deviation was assessed by observing the maximum opening path. Severe malocclusion was assessed by clinical examination, and patients with skeletal malocclusion, Class II and Class III malocclusion, or open bite were excluded.

### Data collection

Data were collected over two months (August–September 2023). Sociodemographic information, including education level (basic schooling or above basic schooling [17]) and employment status (blue-collar or white-collar job [18]), information on preexisting medical or TMJ conditions, dental history and oral hygiene practices, was recorded. Inter examiner reliability was calculated prior to data collection for the plaque score recording (Cronbach’s alpha = 0.84), hand grip strength (Cronbach’s alpha = 0.87) and bite force (Cronbach’s alpha = 0.91).

#### Hand grip strength

Hand grip strength was measured via a JAMAR^®^ hydraulic hand dynamometer [11], a standard evaluation tool for measuring grip strength and squeeze strength [19]. The device was reset to zero to maintain calibration. The participants were educated on the procedure, and accessories from their hands and wrists were removed. Each participant recorded three trials per hand with a 30-second rest between attempts. The average of the highest values was recorded. With both arms naturally lowered and the participant standing, grip strength was assessed [6].

#### Bite force recording

The bite force was recorded using the BYTE Device (©Innovatios Technology), with one reading for each side of every participant and a 30-second interval between each reading [9]. The participants relaxed and seated, and the element was placed on maxillary first or second molar. The participants were instructed to bite with maximum muscle power for three seconds. The instrument measures force in newtons (N) and was autocalibrated with a maximum error of 0.1 N per the manufacturer’s instructions. The average of the highest readings was considered the final bite force reading for both sides.

Standardization for hand grip strength and bite force recording:

In accordance with the guidelines given in both product manuals, the comparison method of standardization was carried out in a step-by-step process among 5 individuals at 2 different time intervals to determine both bite force and grip strength. The point of reference was also documented.

The presence of plaque was recorded via the Plaque Index [20] and the presence or absence of wasting diseases such as attrition, abrasion and erosion was recorded through oral examination.

### Sample size

The sample size was calculated using the software G power 3.1.2. Assuming an effect size of 0.5 at a 95% confidence interval and 80% power, the final sample size was 48.

### Data management and statistical analysis

Data were analysed via SPSS (17.0) version (IBM SPSS^®^ Statistics) -Java (TM) Platform SE binary- IBM Corp: London: UK. The descriptive statistics were tabulated. The Shapiro‒Wilk test was used to assess the normality of the data. For the comparisons of handgrip strength, bite force and plaque score with sociodemographic characteristics, oral health behaviour and wasting diseases, the Mann‒Whitney U test was used. The associations of hand grip strength and bite force with the presence of plaque were assessed via the Spearman correlation coefficient. Rank-based linear regression was then carried out to assess the associations of various independent factors with the accumulation of plaque. The level of statistical significance was set at *p* ≤ 0.05.

## Results

### Demographics and normality testing

A total of 48 patients with a mean age of 39.8 ± 3.2 years visiting the outreach centre and dental clinic participated in this study. Data on demographics, dental visits, oral hygiene practices, wasting diseases, plaque scores, hand grip strength and bite force were recorded. The Shapiro‒Wilk test was used to assess the normality of the data, and the results were statistically significant for the plaque score (W = 0.897, *p* = 0.002), right handgrip strength (W = 0.885, *p* = 0.001), left handgrip strength (W = 0.874, *p* < 0.001), right bite force (W = 0.904, *p* = 0.003) and left bite force (W = 0.911, *p* = 0.005). These results indicated that the data were not normally distributed; hence, nonparametric tests were used for the analysis. The frequency distribution of each parameter is presented in Table 1.

**Table 1:** Frequency table for the variables

		Category	Frequency (Percentage) *N* (%)
**Gender**		Male	20 (41.7)
		Female	28 (58.3)
**Education**		Basic schooling	29 (60.4)
		More than basic schooling	19 (39.6)
**Employment**		Blue collar	20 (41.7)
		White collar	28 (58.3)
**Dental visit**		≤ 6 months	21 (43.8)
		> 6 months	18 (37.5)
		First visit	9 (18.8)
**Reason for visit**		Scaling	3 (6.3)
		Restoration	13 (27.1)
		Extraction	7 (14.6)
		Prosthodontic treatment	5 (10.4)
		Root canal treatment	8 (16.7)
		Orthodontic treatment	3 (6.3)
**Method of brushing**		Horizontal	18 (37.5)
		Horizontal + Vertical	30 (62.5)
**Frequency of brushing**		Once a day	19 (39.6)
		Twice a day	29 (60.4)
**Frequency of changing toothbrush**		Within 3 months	37 (77.1)
		After 3 months	11 (22.9)
**Wasting diseases**	**Attrition**	Present	22 (45.8)
		Absent	26 (54.2)
	**Abrasion**	Present	6 (12.5)
		Absent	42 (87.5)
	**Erosion**	Present	1 (2.1)
		Absent	47 (97.9)
**Plaque Index Score**		Excellent	0 (0)
		Good	36 (75)
		Fair	12 (25)
		Poor	0 (0)

N = Number of participants

### Comparisons of hand grip strength, bite force, and plaque score across demographics

There were 20 male and 28 female participants in this study, and males presented significantly greater handgrip strength and bite force on both sides than did female participants (p value: HG_rt = 0.000, HG_lt = 0.000, BF_rt = 0.010, BF_lt = 0.012). There was no significant difference in the plaque score according to sex.

The participants with basic schooling had significantly greater plaque scores (*p* = 0.034). The employment level of the participants was significantly related to hand grip strength (p value: HG_rt = 0.000, HG_lt = 0.000) and bite force (p value: BF_rt = 0.030, BF_lt = 0.031), indicating greater hand grip strength and bite force among white-collar workers.

### Handgrip strength, bite force, and plaque score in relation to dental visits

The last dental visit of the participants was recorded as ‘6 months or less’ or ‘more than 6 months’ along with the reason for their visit. There was no significant association of the last dental visit with the plaque score, hand grip strength or bite force.

### Comparison of hand grip strength, bite force, and plaque score for oral hygiene practices

All the participants used toothbrushes and toothpaste. A majority [30 (62.5%)] used a combination of horizontal and vertical brushing methods. There was no significant association with plaque score, hand grip strength or bite force. The frequency of brushing was not significantly associated with the plaque score, hand grip strength or bite force. Toothbrush changing frequency was dichotomized as ‘within 3 months’ or ‘more than 3 months’. A significant association was observed between plaque score (p = 0.002) and those changing within 3 months. This group also presented lower hand grip strength and bite force on either side, although these differences were not statistically significant.

### Comparison of hand grip strength, bite force, and plaque score for wasting diseases

Attrition, abrasion and erosion were recorded as present or absent. The participants with attrition had significantly greater handgrip strength on either side (p value: HG_rt = 0.002, HG_lt = 0.016). The means and standard deviations of the variables are given below in Table 2. Comparative results regarding sociodemographics, oral health behaviours and wasting diseases are presented in Table 3a and 3b.

**Table 2:** Descriptive statistics of the variables:

Variable	Mean ± SD
Plaque Score	0.8 ± 0.3
Handgrip strength – right	21.4 ± 9.2 kg
Handgrip strength – left	20.6 ± 8.7 kg
Biteforce – right	15.3 ± 7.1 kg
Biteforce – left	14.9 ± 7.2 kg

Number of participants (N) = 48

**Table 3a:** Comparison of handgrip strength, bite force and plaque score inference according to sociodemographic characteristics, oral health behaviour and wasting diseases

	Sex		Education		Employment status		Last dental visit	
	Z	*p* value	Z	*p* value	Z	*p* value	Z	*p* value
Plaq_score	-0.11	0.992	-2.12	**0.034**	-1.03	0.301	0.26	0.269
HG_rt	-5.47	**0.000**	-0.68	0.500	-3.92	**0.000**	0.14	0.140
HG_lt	-5.11	**0.000**	-1.12	0.264	-3.72	**0.000**	0.25	0.257
BF_rt	-2.58	**0.010**	-2.02	0.043	-2.18	**0.030**	0.08	0.083
BF_lt	-2.52	**0.012**	-1.10	0.273	-2.15	**0.031**	0.31	0.308

Number of participants (N) = 48, Plaq_score = plaque score, HG_rt = handgrip strength right hand, HG_lt = handgrip strength left hand, BF_rt = bite force right side, BF_lt = bite force left side, Z = test value, *p* value = significance (set at ≤ 0.05)

**Table 3b:** Comparison of handgrip strength, bite force and plaque inference according to sociodemographic characteristics, oral health behaviour and wasting diseases

	Method of brushing		Frequency of brushing		Frequency of changing toothbrush		Attrition		Abrasion	
	Z	*p* value	Z	*p* value	Z	*p* value	Z	*p* value	Z	*p* value
Plaq_score	-1.96	0.051	-1.76	0.079	2.37	**0.002**	-1.84	0.108	-1.98	0.056
HG_rt	-0.85	0.394	-1.54	0.124	-1.03	0.215	-3.13	**0.002**	-1.53	0.127
HG_lt	-0.59	0.558	-1.39	0.164	-0.98	0.206	-2.40	**0.016**	-0.89	0.374
BF_rt	-0.38	0.701	-0.59	0.555	-0.57	0.384	-1.67	0.096	-1.43	0.152
BF_lt	-0.90	0.371	-0.84	0.933	-0.94	0.194	-1.88	0.060	-0.44	0.662

Number of participants (N) = 48, Plaq_score = plaque score, HG_rt = handgrip strength right hand, HG_lt = handgrip strength left hand, BF_rt = bite force right side, BF_lt = bite force left side, Z = test value, *p* value = significance (set at ≤ 0.05)

### Correlation analysis

Hand grip strength and bite force were significantly correlated on both sides but not with any of the other variables. Plaque scores were not significantly correlated with handgrip strength or bite force on either the right or left side. Although not significant, the results revealed a negative correlation between the plaque score and handgrip strength and bite force on both sides. Handgrip strength on the right side was positively correlated with handgrip strength on the left side and bite force on both sides. These correlations were statistically significant. The bite forces of both sides were significantly positively correlated (Table 4).

**Table 4:** Correlations between the plaque score, hand grip strength and bite force:

		Plaq_score	HG_rt	HG_lt	BF_rt	BF_lt
Plaq_score	Correlation Coefficient	1.000	-0.032	-0.086	-0.137	-0.056
	*p* value	.	0.831	0.703	0.354	0.703
HG_rt	Correlation Coefficient	-0.032	1.000	0.913	0.481	0.445
	*p* value	0.831	.	**0.000**	**0.001**	**0.002**
HG_lt	Correlation Coefficient	-0.086	0.913	1.000	0.537	0.486
	*p* value	0.559	**0.000**	.	**0.000**	**0.000**
BF_rt	Correlation Coefficient	-0.137	0.481	0.537	1.000	0.875
	*p* value	0.354	**0.001**	**0.000**	.	**0.000**
BF_lt	Correlation Coefficient	-0.056	0.445	0.486	0.875	1.000
	*p* value	0.703	**0.002**	**0.000**	**0.000**	.

Number of participants (N) = 48, Plaq_score = plaque score, HG_rt = handgrip strength right hand, HG_lt = handgrip strength left hand, BF_rt = bite force right side, BF_lt = bite force left side, p value = significance (set at ≤ 0.05)

### Regression analysis

Regression analysis revealed a strong correlation between the variables and the plaque score (*R* = 0.775), with a statistically significant model (F = 2.89, *p* = 0.011). A statistically significant relationship was observed with sex (B = 20.14, *p* = 0.013), with males having a lower rank than females did. The participants who used a combination of horizontal and vertical methods for brushing had significantly lower plaque scores than did those who used the horizontal method alone (B=-6.16, *p* = 0.023). The presence of attrition (B=-15.81, *p* = 0.004) and abrasion (B=-29.05, *p* = 0.001) was significantly associated with a lower plaque score. This is shown in Table 5 below.

**Table 5:** Rank linear regression analysis conducted to predict the plaque score:

	Std. Error	Beta (B)	t	95% Confidence Interval		*p* value
				Lower Bound	Upper Bound	
(Constant)	24.33		4.574	61.199	161.434	0.000
HG_rt	0.417	− 0.274	− 0.686	-1.145	0.573	0.499
HG_lt	0.436	0.801	1.957	− 0.045	1.753	0.062
BF_rt	0.356	− 0.530	-1.656	-1.323	0.144	0.110
BF_lt	0.315	0.511	1.813	− 0.078	1.218	0.082
Sex	7.569	0.646	2.660	4.548	35.727	**0.013**
Education	5.097	0.095	0.543	-7.731	13.262	0.592
Employment	6.017	− 0.397	-1.916	-23.922	0.864	0.067
Last dental visit	4.327	− 0.181	-1.206	-14.130	3.694	0.239
OH_Method	2.542	− 0.404	-2.424	-11.396	− 0.925	**0.023**
OH_Freq_cleaning	5.023	− 0.108	− 0.631	-13.517	7.175	0.534
Frequency of changing brush	5.263	− 0.254	-1.719	-19.886	1.791	0.098
Attrition	5.040	− 0.518	-3.136	-26.185	-5.427	**0.004**
Abrasion	8.085	− 0.612	-3.593	-45.702	-12.399	**0.001**

Number of participants (N) = 48, HG_rt = handgrip strength right hand, HG_lt = handgrip strength left hand, BF_rt = bite force right hand, BF_lt = bite force left hand, OH_Method = oral hygiene method, OH_Freq_cleaning = oral hygiene frequency of cleaning, Std. error = standard error, Beta (B) = standardized coefficient, t = test value, *p* value = significance (set at ≤ 0.05)

## Discussion

The objectives of this cross-sectional study were to assess hand grip strength, bite force, and plaque score individually and the associations among hand grip strength, bite force and plaque score. Among the 48 participants, 20 were male, and 28 were female. No studies have assessed the associations between the factors measured in this study. Few comparisons have been made in different contexts, and such studies are referred to here.

In this study, males presented significantly greater handgrip strength and bite force on both sides than females did. Similar results were reported in a study conducted by Keitaro et al., where younger participants had greater handgrip strength and bite force than older participants did and males outperformed females [9]. Additionally, males had better oral hygiene, as indicated by lower plaque scores in the regression analysis. A paediatric study by Hung-Huey et al. revealed that there was no difference in the plaque score based on sex [13]. Studies have shown that education level is significantly associated with hand grip strength, where one study revealed that individuals with higher education levels had greater handgrip strength [21], and in another study, individuals educated up to high school had greater handgrip strength than other participants did [22]. The results observed in our study were different from those reported in these studies. In a study conducted by Prem et al., lower education was significantly associated with higher plaque scores [23]. No studies in which education was measured against bite force were available. In this study, people with blue-collar jobs had lower hand grip strength, which could be due to overexertion of physical labor causing muscle fatigue, and they also had a lower bite force than people with white-collar jobs did.

Although no studies have compared hand grip strength and bite force with brushing methods and toothbrush replacement frequency, in the present study, both were significantly associated with plaque scores. This is expected, as a proper brushing method is known to reduce the amount of plaque [24], and similarly, in this study, participants who brushed horizontally presented higher plaque scores. A previous trial [25] reported that a longer duration of toothbrush use was associated with a higher plaque level, but this was not the case in our study, where individuals who changed their toothbrush within 3 months presented greater plaque levels. This could be attributed to the improper brushing technique employed as described above or the force of brushing, which was not measured here. The frequency of brushing was compared with hand grip strength in a study conducted among Korean adults, which concluded that men who brushed two times a day had greater handgrip strength than those who brushed once a day [6]. This finding was dissimilar from that of the present study, where no associations were found between the frequency of cleaning, plaque level, handgrip strength and bite force.

Wasting diseases were evaluated in this study, and attrition was significantly associated with only the handgrip strength of both sides, which could indicate that greater forces were probably due to clenching or grinding. No significant association was observed with bite forces for either attrition or abrasion, and a study conducted by Rafael et al. reported a similar result [26]. Both attrition and abrasion were significantly associated with lower plaque score ranks in the regression analysis. There were no studies comparing attrition to plaque levels, whereas a study by Levent et al. revealed that abrasion was significantly associated with less plaque accumulation. This is similar to the findings in this study and could be attributed to the increased unprotected surface available while brushing, which may lead to more wear [27].

Significant correlations were observed between hand grip strength and bite force on both sides in this study. The results of a study by Anton et al. revealed no significant correlation in comparing the right and left sides, but significant results were found for overall comparisons. In a study by Yusuke et al., comparisons were not carried out for both sides separately, and significant results were found for overall comparisons [11, 28]. The plaque score was not significantly correlated with handgrip strength or bite force. A significant association was observed between bite force and plaque score in a study where children with greater bite force presented lower plaque scores [13]. In several studies, reduced handgrip strength was significantly associated with increased plaque levels [29–31], which could be due to its ability to hold and manoeuvre the toothbrush accurately. As of 2024, there were no available studies comparing these parameters.

## Limitations

The present study has certain limitations. First, the cross-sectional design limits the demonstration of causality between handgrip strength, bite force and plaque score. Second, the participants were from urban areas with easy access to dental care and oral behaviour knowledge, which may have led to low plaque scores. Third, the small sample size may limit the generalizability of the findings, reducing the possibility of detecting small associations. Finally, factors such as exercise habits, the hardness of toothbrushes and whether participants were right- or left-handed were not considered. Further studies including these parameters in different geographical locations with more varied samples to assess and expand the findings of this study are recommended.

## Conclusion

The plaque score was significantly associated with education, the method of brushing and the frequency of changing toothbrushes; the handgrip strength and bite force of both the left and right sides were significantly associated with sex and employment status; and the degree of attrition was associated with handgrip strength. Regression analysis revealed significant associations of sex, method of brushing, attrition and abrasion with the plaque score. Within the limitations of this cross-sectional study, no correlations were found between plaque score, hand grip strength and bite force among the participants. Future studies should use larger samples to better represent the population and detect small effects or relationships.

## Supplementary Information

Below is the link to the electronic supplementary material.

Supplementary Material 1

Supplementary Material 2

## Data Availability

The datasets used and/or analyzed in this study are available from the corresponding author upon reasonable request.

## Declarations

### Reporting checklist

The STROBE [32] checklist was used as the reporting guideline for this study. This information has been provided as an additional file along with this manuscript.
