# Supplementary material for: Association of hand grip strength and bite force on the presence of plaque among adults aged 35–44 years in Mangalore – a cross-sectional study
Source: BMC Res Notes. 2025 Aug 6;18:344. doi: 10.1186/s13104-025-07406-w (PMC12326737; doi:10.1186/s13104-025-07406-w)
Supplement: Supplementary file 1 — Supplementary Material 1 [file 13104_2025_7406_MOESM1_ESM.docx]

**CODE BOOK**

Education level:

1 = Basic schooling

2 = Higher than basic schooling

Employment status:

1 = Blue collar jobs

2 = White collar jobs

Last dental visit

1 = 6 months or less

2 = More than 6 months

9 = 1^st^ dental visit

Reason for last dental visit:

1 = scaling

2 = restorations

3 = extraction

4 = prosthodontic treatment

5 = root canal treatment

6 = orthodontic treatment

Method of brushing:

1 = horizontal

2 = vertical

3 = combination of horizontal and vertical

Material used for brushing:

1 = toothbrush and toothpaste

2 = Other aids

Frequency of brushing:

1 = once a day

2 = twice a day

3 = other

Frequency of changing toothbrush:

1 = within 3 months

2 = more than three months

Other aids:

1 = Dental floss

2 = Interdental brush

3 = Mouthwash

Attrition:

1 = Present

2 = Absent

Abrasion:

1 = Present

2 = Absent

Erosion:

1 = Present

2 = Absent
