# Supplementary material for: Association of hand grip strength and bite force on the presence of plaque among adults aged 35–44 years in Mangalore – a cross-sectional study
Source: BMC Res Notes. 2025 Aug 6;18:344. doi: 10.1186/s13104-025-07406-w (PMC12326737; doi:10.1186/s13104-025-07406-w)
Supplement: Supplementary file 2 — Supplementary Material 2 [file 13104_2025_7406_MOESM2_ESM.docx]

**Annexure 1 (Questionnaire - Personal Information)**

1. Serial Number: ____________
2. Date of birth_____________________________________
3. Gender: ________________________
4. Highest level of education you have completed? ______________________
5. Your current employment status **________________________**
6. When was your last dental visit (year/month)? __________________________________
7. The reason for your visit ___________________________________________________
8. Oral Hygiene practices:

Method of cleaning: Horizontal/ vertical/ combination

Materials used: Toothbrush/ toothpaste/ ________________

Frequency of cleaning per day: Once/ twice/ thrice

Frequency of changing toothbrush:

Any other oral hygiene aids used:

**Annexure 2 (Recording form)**

1. Plaque Index: Described by Silness P and Loe H in 1964.

1. Wasting diseases:

Attrition: Present/ Absent

Abrasion: Present/ Absent

Erosion: Present/ Absent

1. Hand Grip Strength Reading:

| Recording 1 | | Recording 2 | | Recording 3 | |  |
| --- | --- | --- | --- | --- | --- | --- |
| Right hand | Left hand | Right hand | Left hand | Right hand | Left hand | Final reading |
|  |  |  |  |  |  |  |

1. Bite Force reading:

| Recording 1 | | Recording 2 | | Recording 3 | |  |
| --- | --- | --- | --- | --- | --- | --- |
| Right side | Left side | Right side | Left side | Right side | Left side | Final reading |
|  |  |  |  |  |  |  |

**Annexure 3 (Information to participants** and **Informed consent form)**

**Title:** “Association of hand grip strength and biting force on presence of plaque among adult population in Mangalore- A cross sectional study.”

**Information to participants**

The purpose of this study is to assess the association of hand grip strength and biting force on presence of plaque among adult population in Mangalore through a cross sectional study. The participants will be initially asked some questions on personal information. Then the hand grip strength and bite force will be recorded and three readings will be taken for each. An intraoral examination will then be carried out which will take a duration of 5 minutes. The information thus obtained can be used to promote oral health and provide better services in future. Participation in the study is entirely on the will of the subject, and he or she can withdraw from the study at any stage. His or her identity will not be revealed and full confidentiality will be assured. This study is for research purpose only. No charges will be applicable for participation in the study.

**Informed consent form**

Name of the participant Date:

Age: ________ Sex: M/F

Address:

(i) I confirm that I have read and understood the information sheet dated ______ for the above study and have had the opportunity to ask questions.

(ii) I understand that my identity will not be revealed in any information released to third parties or published.

(iii) I agree not to restrict the use of any data or photographs or results that arise from this study provided such a use is only for scientific purpose(s).

(iv) I agree for my participation in the above study.

Signature/ thumb impression of participant Signature/ thumb impression of witness

Signature of the principal investigator
